# Loss of STAT6 leads to anchorage-independent growth and trastuzumab resistance in HER2+ breast cancer cells

**DOI:** 10.1371/journal.pone.0234146

**Published:** 2020-06-11

**Authors:** Molly DiScala, Matthew S. Najor, Timothy Yung, Deri Morgan, Abde M. Abukhdeir, Melody A. Cobleigh

**Affiliations:** 1 Division of Hematology, Oncology, and Cell Therapy, Department of Medicine, Rush University Medical Center, Chicago, Illinois, United States of America; 2 Department of Radiation Oncology, University of Kansas Medical Center, Kansas City, Missouri, United States of America; Sechenov First Medical University, RUSSIAN FEDERATION

## Abstract

Approximately 20% of breast cancers are HER2-positive. Trastuzumab has improved patient outcomes significantly for these cancers. However, acquired resistance remains a major hurdle in the clinical management of these patients. Therefore, identifying molecular changes that cause trastuzumab resistance is worthwhile. STAT6 is a transcription factor that regulates a variety of genes involved in cell cycle regulation, growth inhibition, and apoptosis. STAT6 expression is lost in approximately 3% of breast cancers, but little work has been done in the context of trastuzumab resistance in breast cancer. In isogenic cell line pairs, we observed that trastuzumab-resistant cells expressed significantly lower levels of STAT6 compared to trastuzumab-sensitive cells. Therefore, in order to study the consequences of STAT6 loss in HER2+ breast cancer, we knocked out both alleles of the STAT6 gene using somatic cell gene targeting. Interestingly, loss of STAT6 resulted in anchorage-independent growth and changes in several genes involved in epithelial to mesenchymal transition. This study suggests that STAT6 may play a role in the pathophysiology of HER2+ human breast cancer.

## Introduction

Breast cancer is the most common cancer among women. In the United States, it is estimated that 276,480 women will be diagnosed with breast cancer in 2020, and approximately 42,170 additional women will die from their disease [1]. HER2-positive breast cancers account for approximately 20–30% of breast cancers [2]. Historically, HER2-positive breast cancers carried a poor prognosis, but the advent of HER2-targeted therapies significantly improved patient outcomes [3]. However, virtually all patients with metastatic HER2-positive breast cancers treated with these agents develop resistance.

Transcription factors are attractive biomarkers because these proteins have central regulatory roles in gene expression. The STAT6 gene is located on chromosome 12q, and produces a transcription factor [4]. During STAT6 activation, the cytokines interleukin-4 and -13 bind to their associated receptors and cross phosphorylate Janus Kinases (JAK) 1 or 3. This then allows STAT6 to dock to the cytokines and become phosphorylated by JAK proteins [5].

Previous reports have suggested that STAT6 expression can promote apoptosis through increased caspase-3 activity (reviewed in [6]). In breast cancer, up to 18% of breast tumors have decreased or absent STAT6 mRNA expression [7, 8]. Approximately 2% of HER2-positive breast cancers carry mutations in STAT6, which occur sporadically across the gene. The effects of STAT6 loss on breast cancer outcome is unknown. We were interested in exploring the effects of STAT6 loss in the context of trastuzumab resistance in HER2+ breast cancers.

The human breast epithelial cell line MCF-10A is non-tumorigenic and expresses appreciable levels of the STAT6 protein. To explore the functional consequences of STAT6 loss, we knocked out STAT6 in MCF-10A.

MCF-10A cells do not express appreciable levels of the HER2 protein. Therefore, we overexpressed the HER2 protein in parental MCF-10A cells and then knocked out the STAT6 gene. Further analysis revealed that knockout cells formed spheres in liquid culture, consistent with anchorage-independent growth. Quantitation of gene expression patterns associated with anchorage-independent growth was confirmed in STAT-deficient cells. Our findings suggest that loss of STAT6 leads to a more aggressive phenotype and may be one pathway by which cells develop resistance to trastuzumab possibly through the differential expression of STAT6-regulated genes.

## Materials and methods

### STAT6 loss in HER2+ breast cancers

We used data from The Cancer Genome Atlas [9, 10] that was preprocessed by the Broad Institute Genome Data Analysis Center (GDAC) Firehose Tool (**http://gdac.broadinstitute.org/**) and annotated and visualized on the online tool cBioportal [7, 11] to determine the frequency of STAT6 loss in HER2+ breast cancer samples.

### Cell culture

Cells were maintained in a humidified atmosphere, supplemented with 5.1% CO_2_, at 37°C. The non-tumorigenic human breast epithelial cell line MCF-10A (ATCC, Manassas, VA) and its derivatives were grown in DMEM:F12 medium (Life Technologies, Grand Island, NY) devoid of phenol red and supplemented with 5% horse serum (Sigma, Saint Louis, MO), 1% penicillin and streptomycin (Life Technologies, Grand Island, NY), 20 ng/mL epidermal growth factor (EGF; Sigma, Saint Louis, MO), 10 μg/mL bovine serum insulin (Sigma, Saint Louis, MO), 0.5 μg/mL hydrocortisone (Sigma, Saint Louis, MO), and 0.1 μg/mL cholera toxin (Sigma, Saint Louis, MO) (henceforth referred to as “10A supplemented medium”). HER2-overexpressing clones of MCF-10A, referred to as M15, were grown in 10A supplemented medium devoid of EGF and supplemented with 0.4 μg/mL puromycin (Life Technologies, Grand Island, NY; henceforth referred to as “M15 supplemented medium”). BT474 breast cancer cells (ATCC, Manassas, VA) and its derivatives were grown in RPMI-1640 medium (Life Technologies, Grand Island, NY) supplemented with 10% fetal bovine serum (FBS) and 1% penicillin and streptomycin (Life Technologies, Grand Island, NY) (henceforth referred to as “BT474 supplemented medium”). HEK-293T cells (ATCC, Manassas, VA) were grown in Dulbecco’s modified Eagle’s medium (DMEM) supplemented with 5% FBS. Trastuzumab-resistant BT474 cells were grown in BT474 supplemented medium with the addition of 1.0 μM trastuzumab. HEK-293T cells and parental MCF-10A and BT474 cells were purchased from American Type Culture Collection (Manassas, VA). Trastuzumab resistant BT474 and its respective parent line were a generous gift from Dr. Susan Kane (City of Hope, Duarte, CA). Authenticity of these cells was verified using short tandem repeat analysis at the DNA Services Facility at The University of Illinois at Chicago. Table 1 summarizes the different cellular clones used in this study.

**Table 1:** Summary of cellular clones used in this study.

**Parental Line:**	Parental MCF-10A (10A)	
**Child Lines:**		10 STAT6 KO Clone 1 (A1)
		10 STAT6 KO Clone 2 (A2)
**Parental Line:**	HER2-expressing MCF-10A (M15)	
**Child Lines:**		M15 STAT6 KO Clone 1 (M1)
		M15 STAT6 KO Clone 1 (M2)
**Parental Line:**	Parental BT474	
**Child Lines:**		BT474 STAT6 KO Clone 1 (B1)
		BT474 STAT6 KO Clone 1 (B2)
**Parental Line:**	Parental BT474 (trastuzumab sensitive)	
**Child Lines:**		Child BT474 (trastuzumab resistant)

Assays for MCF-10A and M15 cells were conducted in “assay medium,” in which the horse serum in 10A supplemented medium was replaced with 1% charcoal-dextran treated fetal bovine serum (CD-FBS; Fisher Scientific, Pittsburg, PA) and EGF was removed. Assays for BT474 cells were conducted using supplemented BT474 medium. Cells were harvested using TrypLE Express (Life Technologies, Grand Island, NY)

### Lentiviral production and transfection

HEK-293T cells were grown to 40% confluence in antibiotic-free DMEM (Life Technologies, Grand Island, NY) supplemented with 5% FBS (Sigma, Saint Louis, MO). To generate lentivirus, cells were transfected with the plasmids, psPAX2 (12259; Addgene, Cambridge, MA), a lentiviral plasmid containing genetic elements of interest, and VSVg plasmid (8454; Addgene, Cambridge, MA) in a 3:3:1 ratio using Fugene6 Transfection Reagent (Promega, Madison, WI) and Optimem (Fisher Scientific; Waltham, MA) following the manufacturer’s protocol. After 48 hours, the supernatant was harvested, centrifuged to remove cellular debris (300x*g*, 5 minutes), and filtered using a 0.45 μm filter.

### Creation of HER2 overexpressing MCF-10A cells (M15)

A lentiviral plasmid carrying an ERBB2 cDNA in a pBABE-puro backbone (40978; Addgene, Cambridge, MA) was used to generate ERBB2-expressing virus and was used to infect MCF-10A cells to create the M15 cell line. Cells were continually grown in the presence of 0.4 μg/mL puromycin.

### gRNA design

CRISPR-Cas9-mediated NHEJ was used to produce a double stranded break in exon five of *STAT6* between bases 57,106,769 and 57,106,770 (hg38) on chromosome 12q. The guide RNA (gRNA) was designed using the “Optimized CRISPR Design” tool from The Massachusetts Institute of Technology [12]. We ordered the pLentiCRISPR v2 plasmid containing the guide sequence 5′-AGTTTAAGACAGGCTTGCGG-3′ (GenScript, Piscatannay, NJ). This plasmid was used to create lentivirus expressing Cas9 and the above gRNA. Lentivirus-containing supernatant was then concentrated using Lenti-x Concentrator (Clontech, Mountainview, CA) following the manufacturer’s protocol. Concentrated virus supplemented with 8 μg/mL polybrene (Sigma, Saint Louis, MO) was used to infect MCF-10A, M15, and BT474 cells. After 48 hours, cells were selected with either 0.4 μg/mL puromycin for MCF-10A or 2.0μg/ml puromycin for M15 and BT474 cells for three weeks, after which antibiotic selection was discontinued. Putative knockout clones were identified by western blot analysis.

### PCR amplification and Sanger sequencing

Genomic DNA was isolated from cell pellets using DNeasy Blood & Tissue Kit (Qiagen, Germantown, MD) following the manufacturer’s protocol. In order to identify the sequence of each gene targeted allele in the STAT6 knockout (STAT6^-/-^) cells, we PCR-amplified a flanking region of exon 5 in STAT6 and used a combination of Sanger and next generation sequencing. Putative exonic off-target sites predicted by the Optimized CRISPR Design were analyzed by amplifying a flanking region of the putative cut site and analyzed by Sanger sequencing. Oligonucleotides (Integrated DNA Technology, Coralville, Iowa) were designed using Primer-Blast.

PCR was performed using Phusion High Fidelity PCR Master Mix with HF buffer (M0531S; New England Biolabs, Ipswich, MA) using a 3-step protocol per the manufacturers’ instructions (Tables 2 and 3). Annealing temperatures were calculated using the online calculator provided by ThermoFisher [13].

**Table 2:** PCR conditions for amplification of exon 5 of STAT6, off-target regions, and quantitative RT-PCR 96 wells plates.

	Step 1	Step 2				Step 3
		Denaturation	Annealing	Extension	Cycles	
**STAT6 Exon 5 and Off-Target Regions**	98 ^o^C 30 sec	98 ^o^C 10 sec	Shown Below 15 sec	72 ^o^C 20 sec	35	72 ^o^C 5 min
**Prime PCR Assay 96 Well Plates**	95 ^o^C 2 min	95 ^o^C 5 sec	Shown Below 30 sec	-	40	65–95 ^o^C 5 sec/step
	**Annealing Temperature (**^**o**^**C)**					
**STAT6 Exon 5**	63.0					
**CHRNB1**	66.0					
**CDC42BPB**	66.0					
**RP4-671014.6**	67.0					
**STAT6 Exon 5 w/ Illumina adaptors**	69.6					
**Prime PCR Assay**	60					

**Table 3:** Amplifying and sequencing primers.

		Forward Primer	Reverse Primer
**STAT6 Exon *5***	Amplification	AGCAAGGGATAAGGAGCTGA	GCACTTTGACCAAGGTCTCC
	Sequencing	TCACTTCTTGGCATCTGTCCT	TGCAGAGACACTGAGGGTTG
**CHRNBl**	Amplification	TCAGCCCTCTCAGGTCTAGG	CAGTCAACGGGAGGCAGA
	Sequencing	CACATCTGTGTTCCCCTCCT	GAGTAAGAAGCTGAGCCAATGA
**CDC42BPB**	Amplification	GAGGGTCTTGGGTGACAGAA	CATTTTAGGGAGGGGGTCTC
	Sequencing	TGGAAGACTGGCCTGTAACTG	GACTTGGGAGCTCTGAAAGG
**RP4-671014.6**	Amplification	TCTCTCCCATTTCTGCCAAC	GGCGAGAGCGAACAGTTCTT
	Sequencing	AGCCCCTGTTAACCCCTCT	GTGAAGGGGCAGATGACATT
**STAT6 Exon 5 w/ Illumina adaptors (in bold text)**	Amplification	ACACTGACGACATGGTTCTACAAGCAAGGGATAAGGAGCTGA	TACGGTAGCAGAGACTTGGTCTGCACTTTGACCAAGGTCTCC

### Cell proliferation assays

MCF-10A cells and their derivatives were growth arrested prior to beginning growth assays by harvesting with Tryple solution and washing with Hank’s Balanced Salt Solution (HBSS; ThermoFisher Scientific, Waltham, MA) three times. Cells were re-suspended in assay medium. After 24 hours, assay medium was supplemented with 0.2 ng/mL EGF (n = 3). BT474 cells and their clones were suspended in BT474 supplemented medium. Growth proliferation assays in the absence of drug were measured after 24, 48, and 72 hours. Response to trastuzumab was measured by treating cells with or without 1 μM of trastuzumab (Genentech, San Francisco, CA) for 7 days. To visualize differences in cell proliferation, MCF-10A and M15 cells were stained with crystal violet and allowed to dry for 24 hours. After that, 2M acetic acid was added to each well and plates were shaken on an orbital shaker for 20 minutes set at 300 rpm. Then, a 96-well plate spectrophometer was used to measure absorbance of each well. BT474 cells were treated with CellTiter 96® AQ_ueous_ One Solution Cell Proliferation Assay (MTS; Promega, Madison, WI) for 7 days following the manufacturer’s instructions. Measurements were normalized to readings from Day 1 for each cellular clone.

### Western blot analysis

Whole cell protein lysates were made using Laemmli buffer (Biorad Laboratories, Hercules, CA) according to the manufacturer’s instructions. Protein lysates were resolved using NuPage 10% Bis-Tris Gel in MOPS SDS Running Buffer (Life Technologies, Grand Island, NY) for 50 minutes at 200 V. The gel was transferred to a PVDF membrane using NuPage Transfer Buffer (Life Technologies, Grand Island, NY) at 30 V for one hour. The membrane was blocked with solution of 5% nonfat milk in Tris-Buffered Saline solution, supplemented with 0.1% Tween 20 (TBST) for 1 hour and washed with TBST. The membrane was probed with either rabbit anti-STAT6 (ab32520; Abcam, Cambridge, MA) or rabbit anti-GAPDH (51745; Cell Signaling Technology, Inc., Danvers, MA) primary antibodies overnight at 4 ^o^C at a concentration of 1:3000. Membranes were washed three times in TBST and incubated with goat anti-rabbit IgG HRP linked secondary antibody (7074S; Cell Signaling Technology, Inc., Danvers, MA) at a concentration of 1:3000 for one hour and 30 minutes. All antibodies were diluted in TBST supplemented with 5% nonfat milk. Membranes were washed in TBST three times for 20, 5, and 5 minutes. After one final wash in TBS for five minutes, membranes were immediately incubated with Western Lighting Plus ECL (Perkin Elmer, Inc., Waltham, MA) for 2 minutes and used to expose film in a dark room for varying time points from 3 seconds to 20 minutes.

### Sphere formation assay

In order to confirm that cells appearing non-adherent were capable of proliferation in suspension, floating cells were isolated by centrifugation of the growth medium. Cells were then disrupted with tryple and re-suspended in a new flask for three serial passages.

### Quantitative RT-PCR

RNA was extracted from cells and spheres using the RNeasy Mini Kit (Qiagen, Germantown, MD). RNA quantities were measured using a NanoDrop spectrophotometer. RNA was converted to cDNA using SuperScript III First-Strand Synthesis SuperMix for qRT-PCR (ThermoFisher Scientific, Waltham, MA). Quantitative RT-PCR was run on custom 96-well SYBR prime PCR assay plates using SsoAdvanced Universal Sybr Green Super Mix (Biorad Laboratories, Hercules, CA) following the manufacturer’s protocol. Each 96 well plate contained 13 different genes, 2 reference genes, and five different control assays for two different samples as outlined in Table 4. In a separate experiment, levels of STAT6 and ACTB expression were measured in trastuzumab-sensitive and -resistant clones of BT474 cells using predesigned and validated PrimePCR primers were purchased from Biorad (Hercules, CA). Gene expression levels were calculated using the 2^-ΔΔCT^ method and all genes and controls were analyzed in triplicate in each sample.

**Table 4:** Prime PCR custom 96-well plate design for two different samples (white or grey background). Control assays are in bold, and reference genes are underlined.

BMP1	BMP1	BMP1	SMAD7	SMAD7	SMAD7	BMP1	BMP1	BMP1	SMAD7	SMAD7	SMAD7
BMP2	BMP2	BMP2	SNAI1	SNAI1	SNAI1	BMP2	BMP2	BMP2	SNAI1	SNAI1	SNAI1
CDH1	CDH1	CDH1	SNAI2	SNAI2	SNAI2	CDH1	CDH1	CDH1	SNAI2	SNAI2	SNAI2
CDH2	CDH2	CDH2	VIM	VIM	VIM	CDH2	CDH2	CDH2	VIM	VIM	VIM
COL1A2	COL1A2	COL1A2	ACTB	ACTB	ACTB	COL1A2	COL1A2	COL1A2	ACTB	ACTB	ACTB
CTNNB1	CTNNB1	CTNNB1	GAPDH	GAPDH	GAPDH	CTNNB1	CTNNB1	CTNNB1	GAPDH	GAPDH	GAPDH
FN1	FN1	FN1	**gDNA**	**PCR**	**RT**	FN1	FN1	FN1	**gDNA**	**PCR**	**RT**
SMAD1	SMAD1	SMAD1	**RQ1**	**RQ2**	**Empty**	SMAD1	SMAD1	SMAD1	**RQ1**	**RQ2**	**Empty**

### TA cloning

PCR-amplified DNA fragments flanking exon 5 of STAT6 were cloned into the TOPO cloning vector using a TOPO TA Cloning kit (Invitrogen, Carlsbad, California) following the manufacturer’s protocol. Colonies were transformed into chemically competent One Shot Top10 *E*.*coli* (Invitrogen, Carlsbad, California) and 10 individual colonies were selected for each clone. PCR-amplified DNA fragments were sequenced using Sanger sequencing to identify the composition of each individual allele after repair with NHEJ.

### Next generation sequencing

In some clones, the resulting deletion following NHEJ was too large to identify by Sanger sequencing. In these cases, next generation sequencing was used. The cut site region was amplified with flanking primers containing the Illumina sequencing adaptors as shown in Table 3. PCR amplifications were performed as described above and in Table 2. We generated 2x300 reads with the Illumina MiSeq platform at the Center for Genomic Research DNA Services Facility (University of Illinois Chicago). Results were analyzed using CRISPResso to identify the composition of each individual allele after repair with NHEJ [14].

### Statistical analyses

Statistical analyses were performed using a paired, two-tailed Student’s *t*-test or a one-way ANOVA. A *P* value of less than 0.05 was considered statistically significant. Multiple hypothesis testing was corrected using Bonferroni correction [15].

### Ethics statement

No human subject research or animal studies were performed in this study.

## Results

### STAT6 loss in HER2+ breast cancers

Based on the samples analyzed by The Cancer Genome Atlas [9, 10], we observed that STAT6 is lost in 45 (4.1%) patients out of total of 1093 patients across all breast cancer subtypes. In HER2+ breast cancers, STAT6 is lost in 15 (7.6%) out of 197 patients, indicated that it is enriched in this subtype of breast cancer.

### Loss of *STAT6* enhances growth properties of breast cells

**Fig 1:**
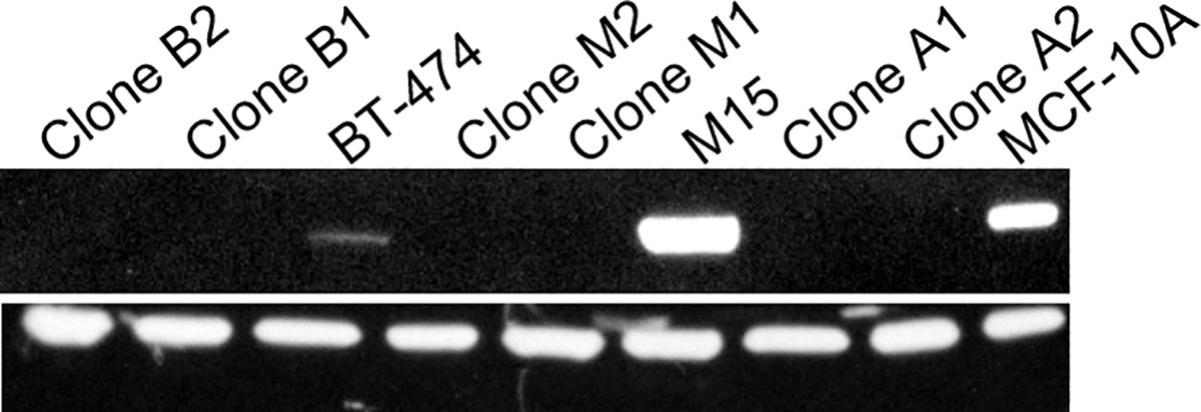
Western blot analysis showing loss of STAT6 protein expression in *STAT6*^*-/-*^ clones. MCF-10A-derived clones A1 & A2, M15-derived clones M1 & M2, and BT474-derived clones B1 & B2.

To isolate the effects of STAT6 loss alone, we started our study by knocking out STAT6 in the human breast epithelial cell line MCF-10A, which expresses appreciable levels of the STAT6 protein (Fig 1 and S1 Fig). Results from all gene targeting studies, including the selection of clones appears in Supplemental Results.

**Fig 2:**
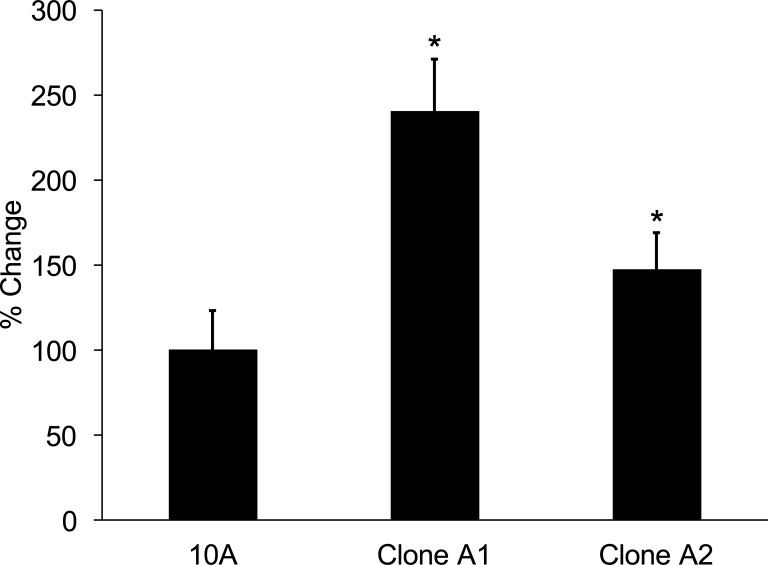
Loss of STAT6 increases growth proliferation rate of clones of MCF-10A cells. * Indicates statistical significance relative to the respective parental cells. Results are the means ± SEM of three independent experiments (n = 3). % Change refers to the change in proliferation relative to the parental cells.

In order to determine how STAT6 loss affects cellular proliferation, we grew parental MCF-10A and their respective STAT6^**-/-**^ clones (Clones A1 and A2) for 72 hours and then counted cell density relative to Day 0. Clones A1 and A2 showed a statistically significant increase in growth compared to parental MCF-10A cells (*p* = 2.14 x 10^**−6**^ and 4.57 x 10^**−7**^, respectively; Fig 2).

### *STAT6* loss enhances growth properties of HER2-expressing breast cells

**Fig 3:**
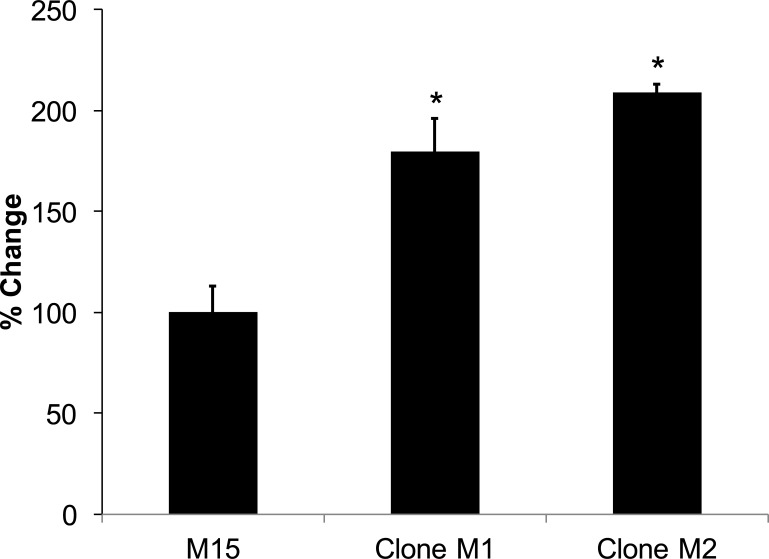
Loss of STAT6 increases growth proliferation rate of clones of M15 cells. * Indicates statistical significance relative to the respective parental cells. Results are the means ± SEM of three independent experiments (n = 3). % Change refers to the change in proliferation relative to the parental cells.

The MCF-10A cell line does not express appreciable levels of the HER2 protein. Therefore, in order to determine the role of STAT6 loss on HER2-expressing breast cells, we expressed the HER2 protein in parental MCF-10A cells, which resulted in M15 cells (Fig 1). Overexpression of HER2 resulted in a 80.9% (n = 4, SD = 6.09) increase in cellular proliferation after seven days, similar to the results reported by others [16]. We then knocked out STAT6 in M15 cells, which resulted in clones M1 and M2. When these knock-outs were grown for 72 hours, clones M1 and M2 showed a statistically significant increase in growth compared to parental M15 cells (*p* = 8.6 x 10^−4^ and 1.5 x 10^−3^, respectively; Fig 3).

### Loss of *STAT6* enhances growth properties of HER2-expressing breast cancer cells

**Fig 4:**
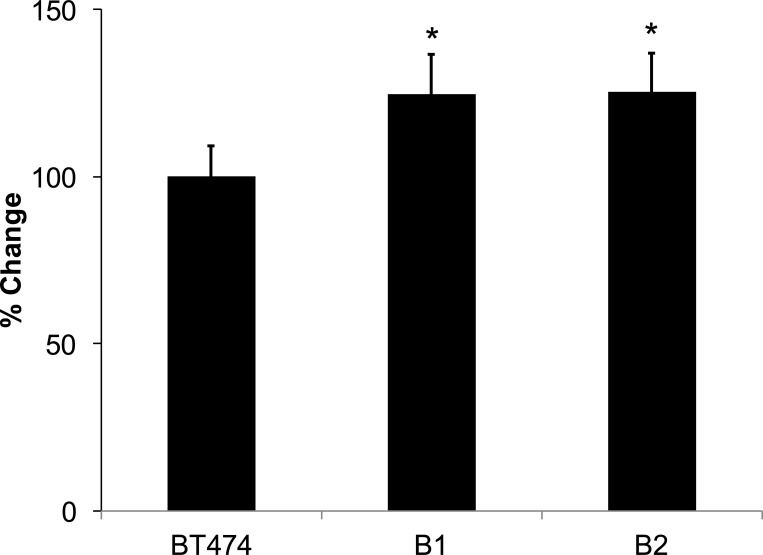
Loss of STAT6 increases growth proliferation rate of clones of BT474 cells. * Indicates statistical significance relative to the respective parental cells. Results are the means ± SEM of three independent experiments (n = 3). % Change refers to the change in proliferation relative to the parental cells.

The MCF-10A cell line is not derived from a breast cancer. In order to determine the role of STAT6 loss on HER2-expressing breast cancer cells, we knocked out STAT6 in the human breast cancer cell line BT-474 to generate clones B1 and B2. When these knock-outs were grown for 72 hours, they showed a statistically significant increase in growth compared to parental BT-474 cells (*p* = 0.046 and 0.039, respectively; Fig 4).

### Loss of *STAT6* increases growth rate and cell viability in the presence of trastuzumab

**Fig 5:**
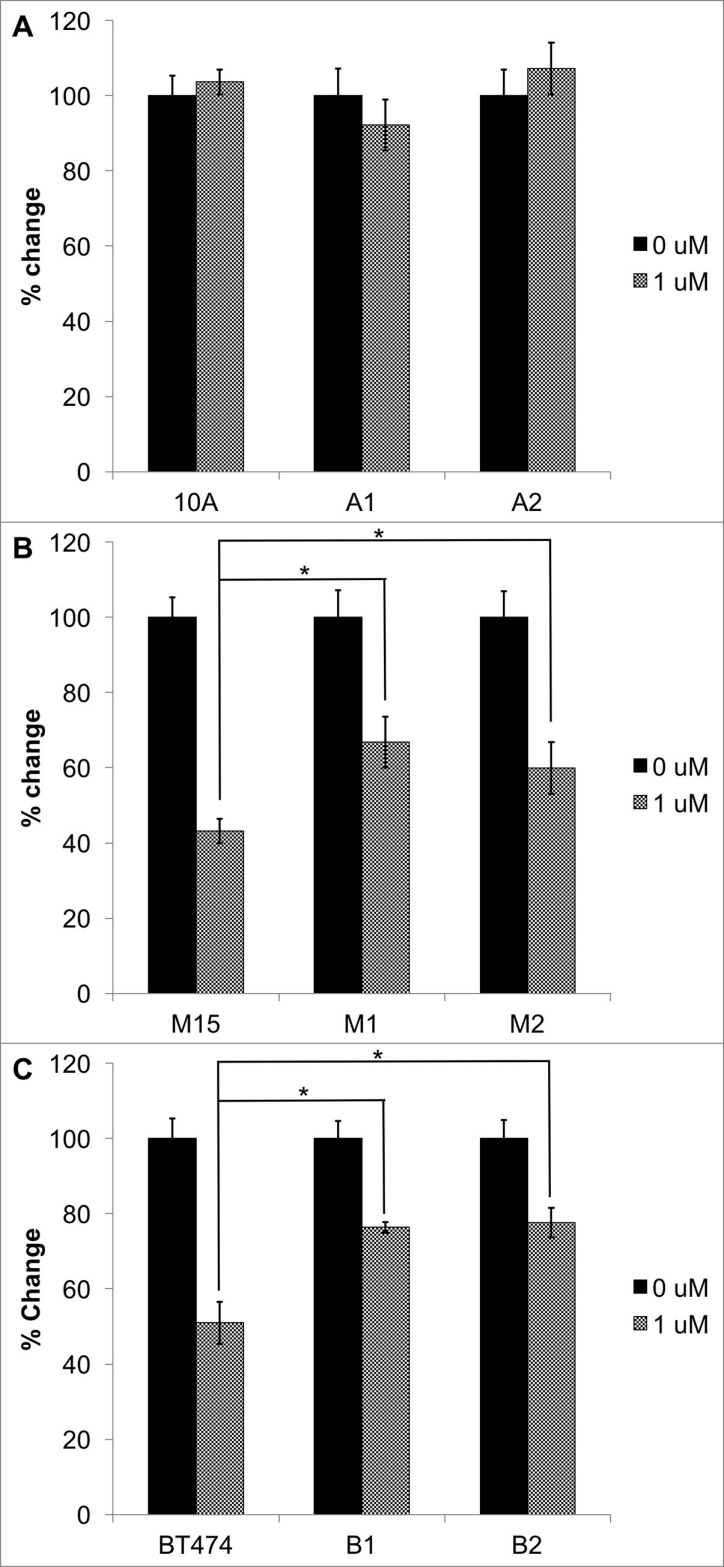
STAT6 null clones of (A) MCF-10A and (B) M15 and (C) BT474 were treated with or without 1uM trastuzumab for 7 days. *Indicates statistical significance relative to the respective parental cell response. Results are the means ± SEM of three independent experiments (n = 3).

We were interested in determining if STAT6 loss in HER2+ breast cancers could contribute to trastuzumab resistance. Therefore, we measured growth of STAT6^-/-^ clones compared to parental controls for seven days in the presence or absence of 1μM trastuzumab. Parental MCF-10A cells do not express appreciable levels of ERBB2 and as expected, growth of these cells and their STAT6 knock-outs A1 and A2 were unaffected by trastuzumab (Fig 5A). However, loss of STAT6 conferred significant resistance to trastuzumab in M15 knock-out clones M1 and M2 (*p* = 3.00 x 10^−4^ and 0.044, respectively; Fig 5B). The same phenomenon was observed in BT474 clones B1 and B2 compared with their parental cells BT474 cells (*p* = 5.41 x 10^−4^ and 9.66 x 10^−5^, respectively; Fig 5C).

### Loss of *STAT6* promotes sphere growth and an EMT phenotype in *STAT6*^-/-^ clones and spheres

We observed that all STAT6^-/-^ clones grew as adherent cells and non-adherent spheres (S2 Fig), suggesting anchorage-independence. In order to confirm that these non-adherent cells were capable of proliferation, floating cells were isolated, disrupted with Tryple and then re-suspended in new flasks for three serial passages. We continued to observe proliferating adherent and non-adherent cells after each passage.

Anchorage-independent growth is associated with epithelial to mesenchymal transition (EMT). Therefore, we measured the mRNA expression of 13 EMT-related genes in all of the adherent clones, spheres, and parental cells using quantitative RT-PCR. We examined the following EMT genes: bone morphogenetic protein-1 (BMP1), bone morphogenetic protein-2 (BMP2), epithelial cadherin (CDH1), neural cadherin (CDH2), collagen type I alpha 2 chain (COL1A2), catenin beta-1 (CTNNB1), fibronectin-1 (FN1), SMAD family member-1 (SMAD1), SMAD family member-7 (SMAD7), snail family transcriptional repressor-1 also known as snail (SNAI1), snail family transcriptional repressor-2 also known as slug (SNAI2), tetraspanin-13 (TSPAN13), and vimentin (VIM). Gene expression was normalized to the expression of the housekeeping genes GAPDH and ACTINB.

**Fig 6:**
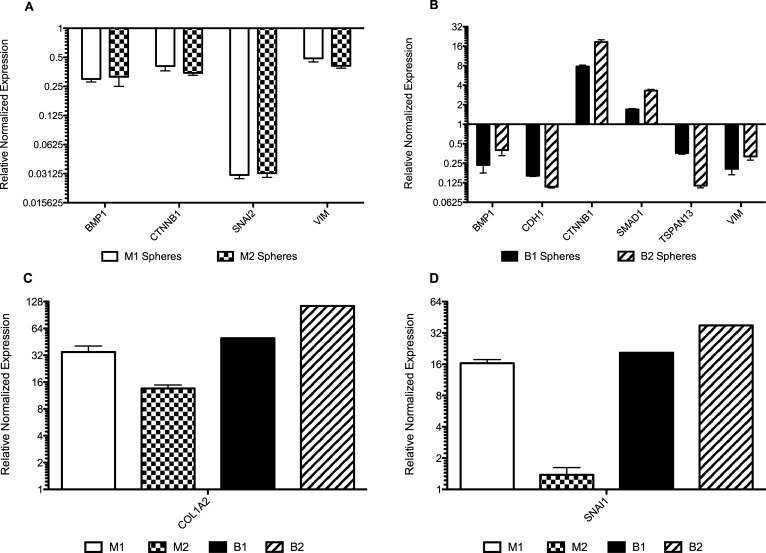
(A) Gene expression from quantitative RT-PCR results of M1 and M2 spheres compared to their respective adherent cells. BMP1, CTNNB1, SNAI2, and VIM were downregulated, while COL1A2 upregulated in both clones. (B) Gene expression results from quantitative RT-PCR results of B1 and B2 spheres compared to their respective adherent cells. COL1A2, CTNNB1, SMAD1, and SNAI1 are significantly upregulated in spheres, while BMP1, CDH1, TSPAN13, and VIM are significantly downregulated. Expression of COL1A2 (C) and SNAI1 (D) were plotted alone for clarity due to the large scale of the increase. SNAI1 was not significantly upregulated in clone M2.

First, we compared the gene expression of the 13 EMT-related genes in the STAT6^-/-^ sphere populations from HER2+ cells compared with their respective adherent STAT6^-/-^ populations. Significantly differentially expressed genes are shown in Fig 6. We found that BMP1, TSPAN13, and VIM were significantly downregulated, while COL1A2 was significantly upregulated in all STAT6^-/-^ HER2+ spheres relative to their respective parental STAT6^+/+^ clones and chose these genes for further analyses.

**Fig 7:**
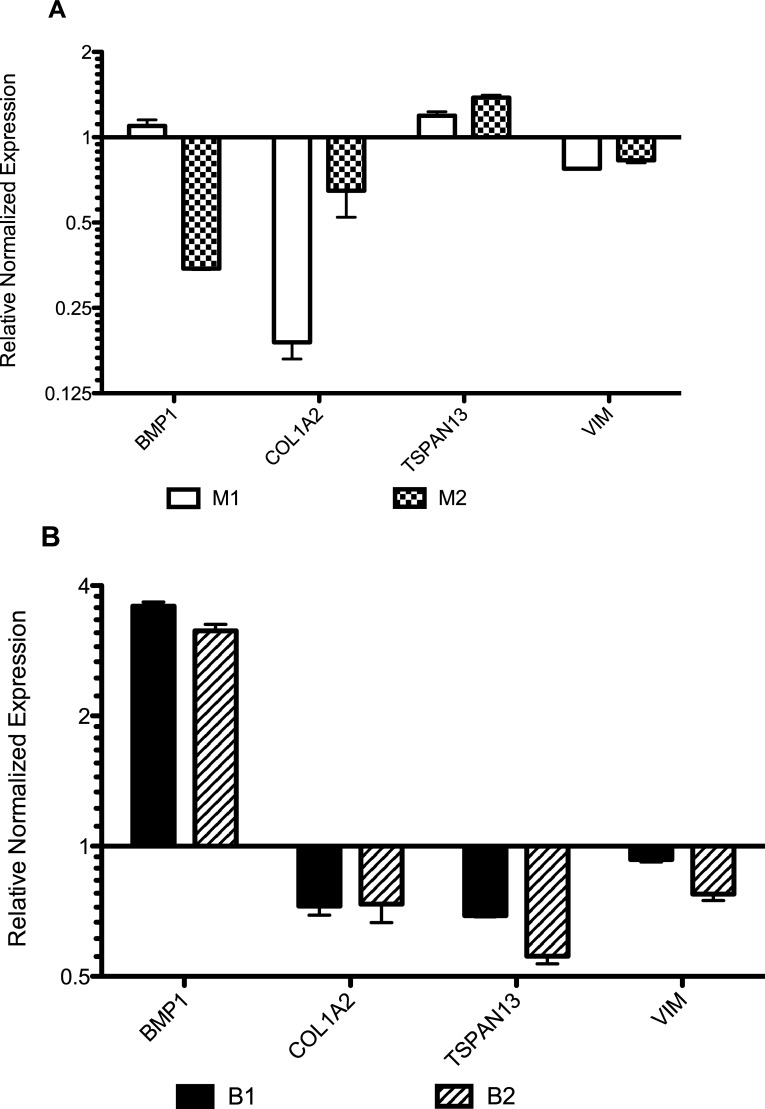
Gene expression results from quantitative RT-PCR results of (A) M1 and M2 and (B) B1 and B2 adherent cells compared to their respective STAT6^+/+^ parental cells.

Statistically-significant changes in gene expression in Fig 6 may be associated with either i) STAT6 loss, ii) anchorage-independent growth, or iii) both STAT6 loss and anchorage-independent growth. We wanted to identify the differentially expressed genes that were associated with anchorage-independent growth. Therefore, we calculated the changes in gene expression in BMP1, COL1A2, TSPAN13, and VIM the adherent STAT6^-/-^ clones compared to their respective parental STAT6^+/+^ cell lines (Fig 7). We observed that BMP1 was downregulated in clones of M15 (M1 and M2), but upregulated in clones of BT474 (B1 and B2). These discordant results make it difficult to speculate on the role of this gene in STAT6 loss. TSPAN13 and VIM were downregulated in all adherent STAT6^-/-^ clones of M15 and BT474. Therefore, TSPAN14 and VIM are downregulated following STAT6 loss and are further downregulated with sphere formation. Finally, COL1A2 was significantly downregulated in all adherent STAT6^-/-^ clones of M15 and BT474 (Fig 7), but highly upregulated in STAT6^-/-^ clones growing as spheres. COL1A2 was the only gene whose expression was concordant across all adherent STAT6^-/-^ clones and was uniquely changed in STAT6^-/-^ sphere clones. i.e., COL1A2 was significantly downregulated in all adherent STAT6^-/-^ clones and significantly upregulated in all STAT6^-/-^ sphere clones.

Sphere formation is associated with anchorage-independent growth, which is associated with a more aggressive cancer phenotype. Taken together, this suggests that an increase in COL1A2 expression may be necessary for sphere formation and that inhibition of COL1A2 could cause cells to revert to a less aggressive phenotype.

### Trastuzumab resistance leads to loss of *STAT6*

We measured STAT6 expression in parental BT474 cells (STAT6 intact) and a clone that was conditioned to grow in the presence of trastuzumab (trastuzumab resistance). qPCR revealed that the expression of STAT6 in the parental BT474 cells was 1.02 (n = 3; SD = 0.07) and in the trastuzumab-resistant clone was 0.39 (n = 3; SD = 0.04). The relative normalized expression of STAT6 in the trastuzumab-resistant clone compared to the parent clone was 0.38 (n = 3; SD = 0.04; p = 0.000135).

## Discussion

In this study, we explored the effect of STAT6 loss on trastuzumab resistance in breast cancer cell lines. In order to isolate the effects of STAT6 loss, we created isogenic cell lines by knocking out both copies of STAT6 in the human breast epithelial cell line MCF-10A, which has two wild-type copies of HER2 using the CRISPR/Cas9 system. Isogenic cellular clones differ in the genotype of a small number of genes (in the case of our study, only one gene). This allowed us to isolate the effects of STAT6 loss. Isogenic cells provide a useful experimental platform for understanding complex diseases like HER2-positive breast cancer, which harbor many different genetic aberrations [17].

The MCF-10A cell line is a non-tumorigenic cell line that carries a normal chromosome number, is devoid of any known oncogenic mutations and requires several growth factors to proliferate. Moreover, it is chromosomally stable and was not derived from a cancer patient, so these cells have never been exposed to DNA-damaging chemotherapeutic agents. However, the MCF-10A cell line does not express appreciable levels of the HER2 gene; therefore, we created the HER2-overexpressing clone of MCF-10A, M15. Parental MCF-10A and M15 cells express high levels of the STAT6 protein (Fig 1), making these cells ideal models for studying the phenotype associated with STAT6 loss. Therefore, we deleted STAT6 in parental MCF-10A cells and M15 cells (HER2-expressing MCF-10A cells). The isogenic nature of MCF-10A clones allowed us to isolate the effects of STAT6 loss alone in the context of HER2-expressing breast cells. The use of parental MCF-10A cells (non HER2-expressing) allowed us to determine if the phenotype observed in STAT6 null M15 cells was due to HER2 expression, STAT6 loss, or both. Finally, we also deleted STAT6 in the HER2-over-expressing breast cancer cell line BT474 in order to determine if our findings were also applicable to HER2-amplified breast cancer cells.

It is important to note that loss of STAT6 expression appears to improve the growth properties of HER2-negative cells also, as clones A1 and A2 grew faster than parental MCF-10A cells (Fig 2). This is not surprising, as there are many examples of genes that affect drug response across different breast cancer subtypes. For example, oncogenic mutations in PIK3CA lead to increased cellular proliferation and altered cellular signaling [18]. Current professional guidelines now recommend that ER-positive breast cancer patients with pathogenic mutations in PIK3CA receive the drug combination alpelisib plus fulvestrant. This recommendation is based upon results from the SOLAR-1 trial, which showed alpelisib plus fulvestrant improved median progression-free survival from 5.7 months to 11.0 months in ER-positive, HER2-negative, advanced or metastatic breast cancer whose disease had progressed or recurred on or after an aromatase inhibitor-based treatment. Similarly, clinical studies in HER2-positive breast cancers have found that patients with mutations in PIK3CA experience decreased benefit from neoadjuvant trastuzumab [19, 20]. Thus, mutant PIK3CA is an example of an altered gene that is associated with changes in drug responses across multiple subtypes of breast cancer.

We were interested in exploring how STAT6 loss affects trastuzumab response. Therefore, we tested the effects of trastuzumab treatment on STAT6 null-cells compared to their respective STAT6-expressing parent clones. We observed that STAT6^-/-^ clones from both M15 and BT474 cell lines were less sensitive to trastuzumab than parental, STAT6-expressing cells. Our results suggest that STAT6 loss may contribute to trastuzumab resistance in HER2-positive breast cancer cells.

Our initial results suggest that loss of STAT6 expression is associated with an aggressive growth phenotype. 1) We observed that STAT6 null cells grew faster than their parental STAT6-expressing parental clones. 2) Clones devoid of STAT6 expression were anchorage independent in vitro; as cells grew in two populations, i.e., adherent and floating (parental MCF-10A, M15, and BT474 cells with expression of STAT6 grow as adherent cells). Loss of STAT6 appears to be associated with trastuzumab resistance because we observed a highly significant decrease in STAT6 expression in clones of BT474 that were conditioned to be resistant to trastuzumab. i.e., STAT6 expression decreased after the cells acquired resistance to trastuzumab. Taken together, we hypothesize that STAT6 loss may contribute to a worse prognosis in HER2-positive patients by contributing to trastuzumab resistance and EMT.

EMT is the biologic process that occurs when epithelial cells undergo mesenchymal transition. EMT is an essential component of normal embryogenesis, organ development, tissue remodeling, and wound repair [21]. However, aberrant activation of EMT is considered a pre-requisite for solid tumor metastasis and cancer progression. Some phenotypic markers of EMT include enhanced migratory capability, invasiveness, increased resistance to apoptosis, and increased production of extracellular matrix components [22]. We observed the formation of cellular spheres that was consistent with anchorage-independent growth. Several studies have reported correlations between the expression of EMT-related genes and anchorage-independent growth [23]. Therefore, we explored the expression of 13 EMT-related genes in the STAT6^-/-^ clones used in our study.

The spheres from STAT6^-/-^ clones derived from BT474 and M15 cells shared overexpression of COL1A2 and SNAI1, which we found to be significant because they are commonly upregulated during EMT. Interestingly, these were the only two genes commonly upregulated in clones of M15 and BT474 and they were also upregulated by an order of magnitude. Although there were several genes downregulated in STAT6 null clones of M15 and BT474, these genes were also downregulated in parental M15 cells (STAT6-expressing) when compared to parental MCF-10A cells (STAT6-expressing and low levels of HER2). This suggests that the introduction of the HER2 gene into MCF-10A cells to create M15 cells led to downregulation of these genes. In the current study, we were interested in changes associated with STAT6 loss in HER2-overexpressing cells. Therefore, these downregulated genes were not studied further.

COL1A2 is a component of the extracellular matrix (ECM), but is also observed in many breast and ovarian cancer drug-resistant cell lines, which indicates that it can play an important role in drug resistance at the tissue and cellular level [24]. COL1A2 was found to be differentially upregulated in breast cancer cells compared to normal breast tissue [25]. Higher levels of type 1 collagen have been observed in metastatic lesions of individuals with metastatic disease [26]. High COL1A2 mRNA expression was positively correlated with poor overall survival time in patients with gastric cancer [27]. In ovarian cancer, overexpression of COL1A2 has been associated with resistance to several chemotherapies by decreasing apoptosis [24]. In our study, we found that adherent STAT6^-/-^ cells expressed less COL1A2 than their respective parental STAT6^+/+^ clones. However, STAT6^-/-^ spheres expressed significantly more COL1A2 than their respective adherent STAT6^-/-^ clones. These findings suggest that increased expression of COL1A2 in the STAT6^-/-^ clones may represent a subset of drug resistant cells that do not respond to trastuzumab.

We observed a significant decrease in BMP1, TSPAN13, and VIM. Others have found that EMT is associated with increased expression of these genes [28]. The reasons for this are unclear and require further investigation. However, our findings regarding COL1A2 are in agreement with previous studies implicating STAT6 expression in driving apoptosis [24] and thus loss of STAT6 would prevent apoptosis. Others have found that STAT6 loss is a negative prognostic marker in HER2-positive breast cancers [29].

Finally, we observed that a clone of BT474 cells that was conditioned to be resistant to trastuzumab lost expression of STAT6. STAT6 resides on chromosome 12. Others have reported 27 potential intra-chromosomal translocations involving chromosome 12 of the BT474 cell line by using RNAseq [30], which implies that chromosome 12 may be susceptible to genetic alterations. Rondón-Lagos et al. reported that BT474 cells have a modal chromosome number near tetraploid [31]. This suggests that BT474 cells have a high level of aneuploidy. Taken together, these translocations and aneuploidy may explain how BT474 cells are able to adapt to environmental challenges, i.e., drug selection with trastuzumab, and may explain how expression of STAT6 was lost in these cells.

To conclude, our data suggests that loss of STAT6 results in EMT and trastuzumab resistance. These results warrant further research into the role of STAT6 in patients with HER2-positive breast cancer. Our findings suggest that inhibition of COL1A2 may be a potential therapeutic strategy in breast cancer; although the ubiquitous expression of this protein may present some challenges. As genotyping technology becomes more advanced and cost effective, data from gene studies will reveal more important drug-gene interactions. This will help us pursue the ultimate goal of personalizing patient drug treatment to achieve the best therapeutic outcome.

## Supporting information

S1 Fig Unadjusted western blot image. This is the unadjusted image used in Fig 1 (see file “S1 Raw Image”). Multiple exposures of the gel with varying exposure times appear in each quadrant.
(DOCX)

S2 Fig Representative images of sphere formation in STAT6 null clones. Left panel shows spheres derived from the STAT6^-/-^ parental MCF-10A clone (Clone A2), while the panel on right shows spheres derived from the STAT6^-/-^ M2 clone.
(JPG)

S3 Fig *In silico* results of Sanger sequencing of *STAT6* knockout clones. Clone A1 and A2 compared to MCF-10A parent, clone M1 and clone M2 compared to M15 parent, and clone B1 and clone B2 compared to BT474. Parental MCF-10A, M15, and BT474 share the same sequence as the reference listed above. The gRNA target region is depicted in bold. The PAM sequence is shown in red typeface. Indels are highlighted in red, insertion sequences are highlight in blue, new stop codons are highlighted in yellow.
(DOCX)

S4 Fig Agarose gel images of 1kb and 2kb PCR products flanking the STAT6 cut site to confirm homozygous deletion. Clone A2 was loaded in lane 1. MCF-10A was loaded into lane 2 and was used as a control. Following a CRISPR-mediated double-strand break in both copies of STAT6, NEJM repairs the break and inserts a random indel. Each allele should have a unique indel resulting in alleles of different sequences and lengths. Thus, the presence of a single band suggests that M2 contains a homozygous deletion.
(DOCX)

S5 Fig Electropherograms depicting possible off-target sites of Cas9 endonuclease activity in STAT6-/- clones. Using the CRISPR Design Tool at the Broad Institute, we were able to identify putative off target regions in the genes above. Primers flanking the putative off-target sites were used to amplify regions in CHRONB1, RP4-671014.6, and CDC42BPB, which were then analyzed via Sanger sequencing. All STAT6-/- clones did not exhibit any mutations, indicating that no off-targeting was present in these clones.
(DOCX)

S1 File (DOCX)

S1 Raw Image (PDF)
