# Supplementary material for: Loss of STAT6 leads to anchorage-independent growth and trastuzumab resistance in HER2+ breast cancer cells
Source: PLoS One. 2020 Jun 11;15(6):e0234146. doi: 10.1371/journal.pone.0234146 (PMC7289443; doi:10.1371/journal.pone.0234146)

**Supplemental Figure 1. Unadjusted western blot image.** This is the unadjusted image used in Figure 1 (see file “S1_raw-image.pdf”). Multiple exposures of the gel with varying exposure times appear in each quadrant.


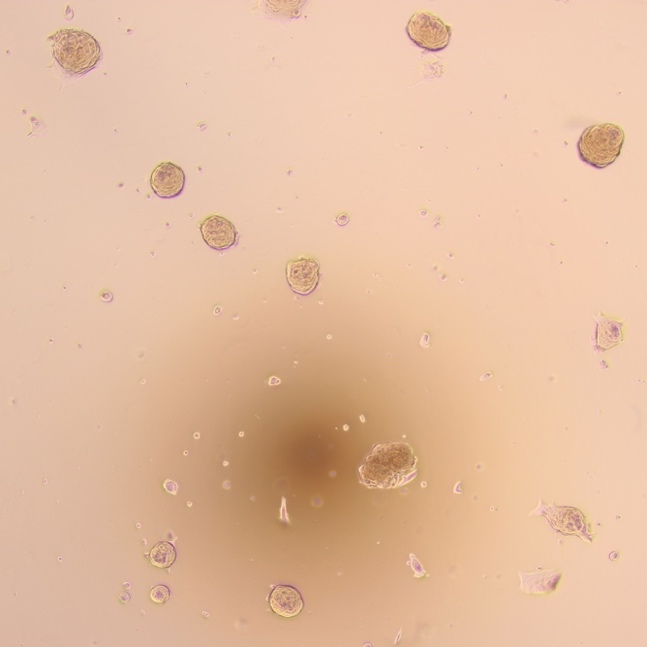

Supplement: S1 Fig — This is the unadjusted image used in Fig 1 (see file “S1 Raw Image”). Multiple exposures of the gel with varying exposure times appear in each quadrant. (DOCX) [file pone.0234146.s001.docx]
