# Supplementary figures and images for: Loss of STAT6 leads to anchorage-independent growth and trastuzumab resistance in HER2+ breast cancer cells
Source: PLoS One. 2020 Jun 11;15(6):e0234146. doi: 10.1371/journal.pone.0234146 (PMC7289443; doi:10.1371/journal.pone.0234146)

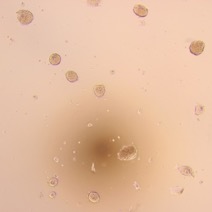

Supplement: S2 Fig — Left panel shows spheres derived from the STAT6-/- parental MCF-10A clone (Clone A2), while the panel on right shows spheres derived from the STAT6-/- M2 clone. (JPG) [file pone.0234146.s002.jpg]
