# Supplementary material for: Loss of STAT6 leads to anchorage-independent growth and trastuzumab resistance in HER2+ breast cancer cells
Source: PLoS One. 2020 Jun 11;15(6):e0234146. doi: 10.1371/journal.pone.0234146 (PMC7289443; doi:10.1371/journal.pone.0234146)

**Supplemental Figure 3.** ***In silico* results of Sanger sequencing of *STAT6* knockout clones.** Clone A1 and A2 compared to MCF-10A parent, clone M1 and clone M2 compared to M15 parent, and clone B1 and clone B2 compared to BT474. Parental MCF-10A, M15, and BT474 share the same sequence as the reference listed above. The gRNA target region is depicted in bold. The PAM sequence is shown in red typeface. Indels are highlighted in red, insertion sequences are highlight in blue, new stop codons are highlighted in yellow.


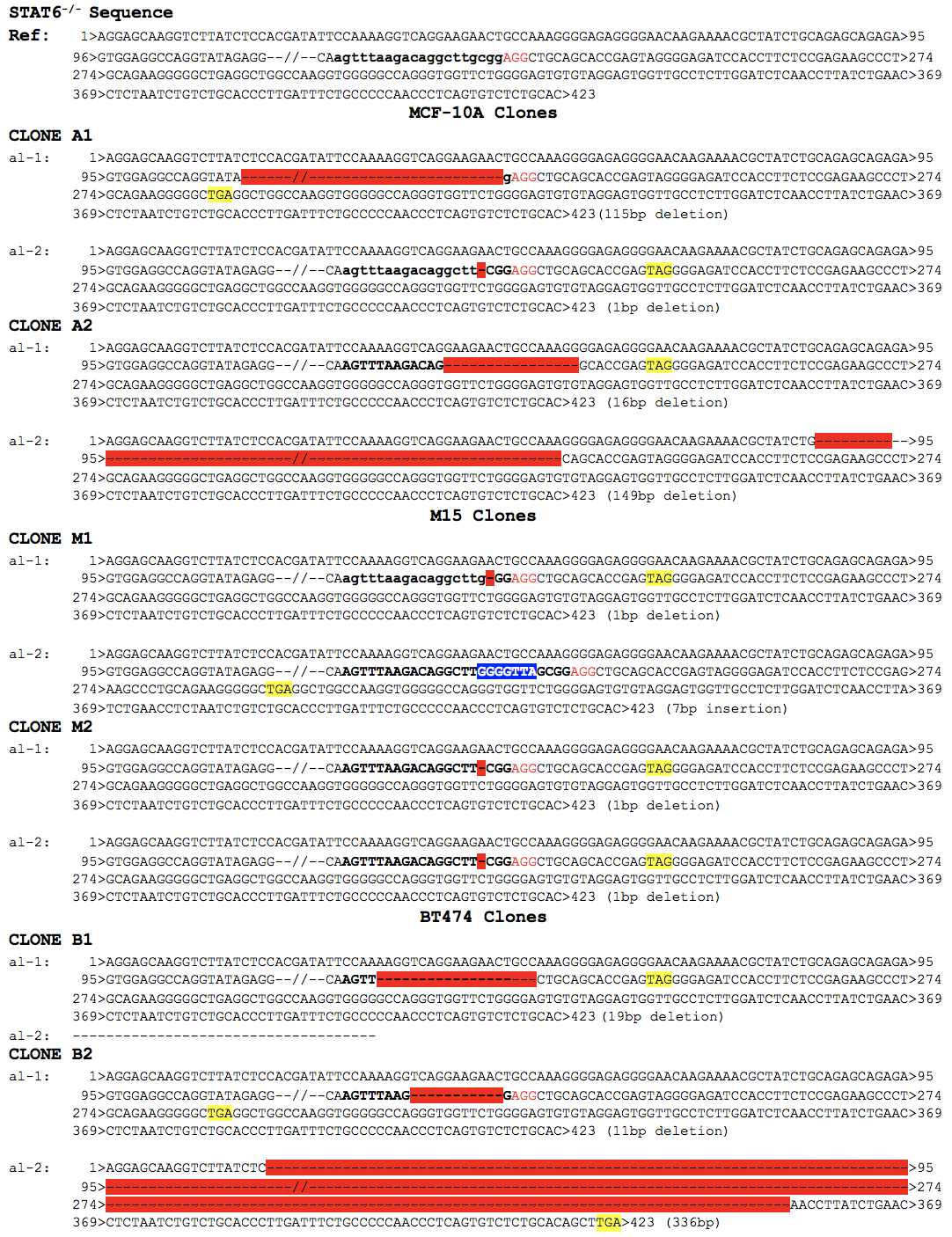

Supplement: S3 Fig — Clone A1 and A2 compared to MCF-10A parent, clone M1 and clone M2 compared to M15 parent, and clone B1 and clone B2 compared to BT474. Parental MCF-10A, M15, and BT474 share the same sequence as the reference listed above. The gRNA target region is depicted in bold. The PAM sequence is shown in red typeface. Indels are highlighted in red, insertion sequences are highlight in blue, new stop codons are highlighted in yellow. (DOCX) [file pone.0234146.s003.docx]
