# Supplementary material for: Loss of STAT6 leads to anchorage-independent growth and trastuzumab resistance in HER2+ breast cancer cells
Source: PLoS One. 2020 Jun 11;15(6):e0234146. doi: 10.1371/journal.pone.0234146 (PMC7289443; doi:10.1371/journal.pone.0234146)

**Supplemental Figure 4. Agarose gel images of 1kb and 2kb PCR products flanking the STAT6 cut site to confirm homozygous deletion.** Clone A2 was loaded in lane 1. MCF-10A was loaded into lane 2 and was used as a control. Following a CRISPR-mediated double-strand break in both copies of STAT6, NEJM repairs the break and inserts a random indel. Each allele should have a unique indel resulting in alleles of different sequences and lengths. Thus, the presence of a single band suggests that M2 contains a homozygous deletion.

**M2 MCF10A**

**1kb PCR Product**


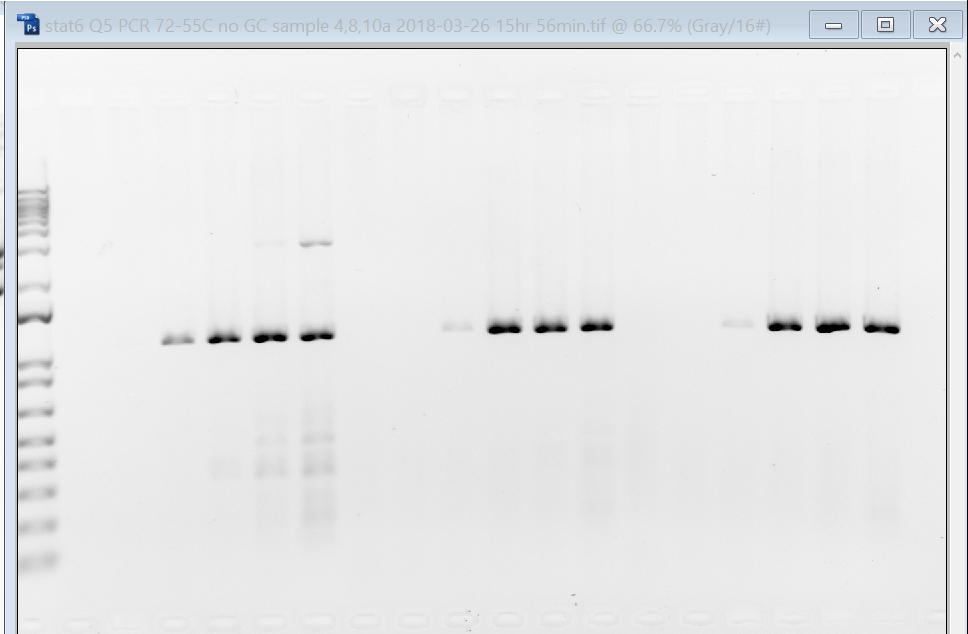

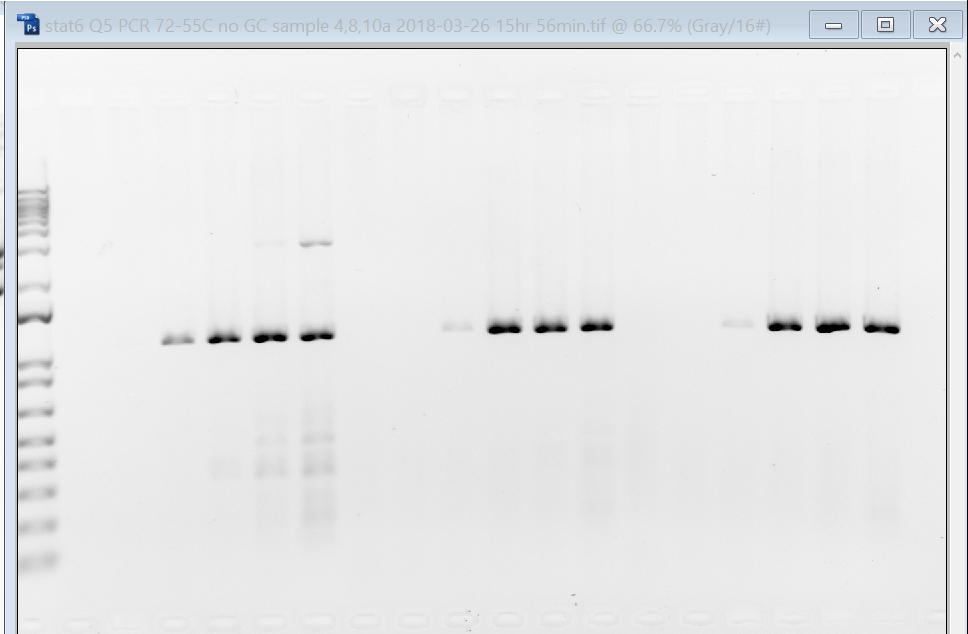


**M2 MCF10A**

**2kb PCR Product**


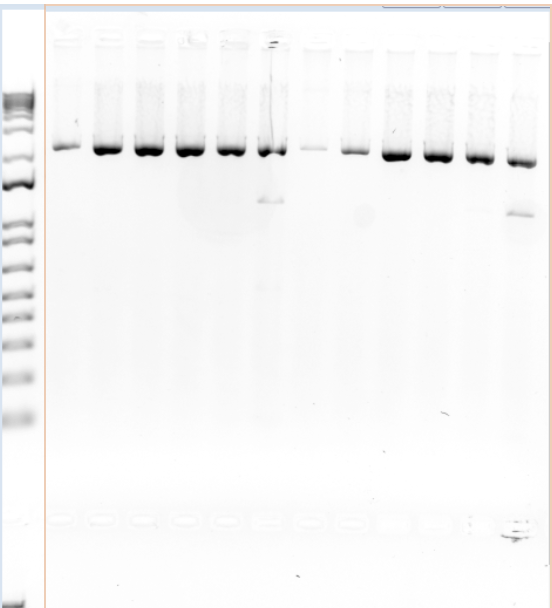

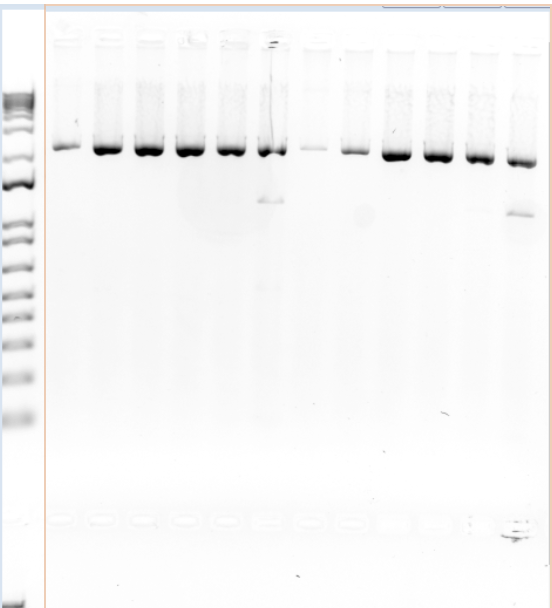

Supplement: S4 Fig — Clone A2 was loaded in lane 1. MCF-10A was loaded into lane 2 and was used as a control. Following a CRISPR-mediated double-strand break in both copies of STAT6, NEJM repairs the break and inserts a random indel. Each allele should have a unique indel resulting in alleles of different sequences and lengths. Thus, the presence of a single band suggests that M2 contains a homozygous deletion. (DOCX) [file pone.0234146.s004.docx]
