# Supplementary material for: Loss of STAT6 leads to anchorage-independent growth and trastuzumab resistance in HER2+ breast cancer cells
Source: PLoS One. 2020 Jun 11;15(6):e0234146. doi: 10.1371/journal.pone.0234146 (PMC7289443; doi:10.1371/journal.pone.0234146)

**Supplemental Figure 5. Electropherograms depicting possible off-target sites of Cas9 endonuclease activity in STAT6-/- clones.** Using the CRISPR Design Tool at the Broad Institute, we were able to identify putative off target regions in the genes above. Primers flanking the putative off-target sites were used to amplify regions in CHRONB1, RP4-671014.6, and CDC42BPB, which were then analyzed via Sanger sequencing. All STAT6-/- clones did not exhibit any mutations, indicating that no off-targeting was present in these clones.


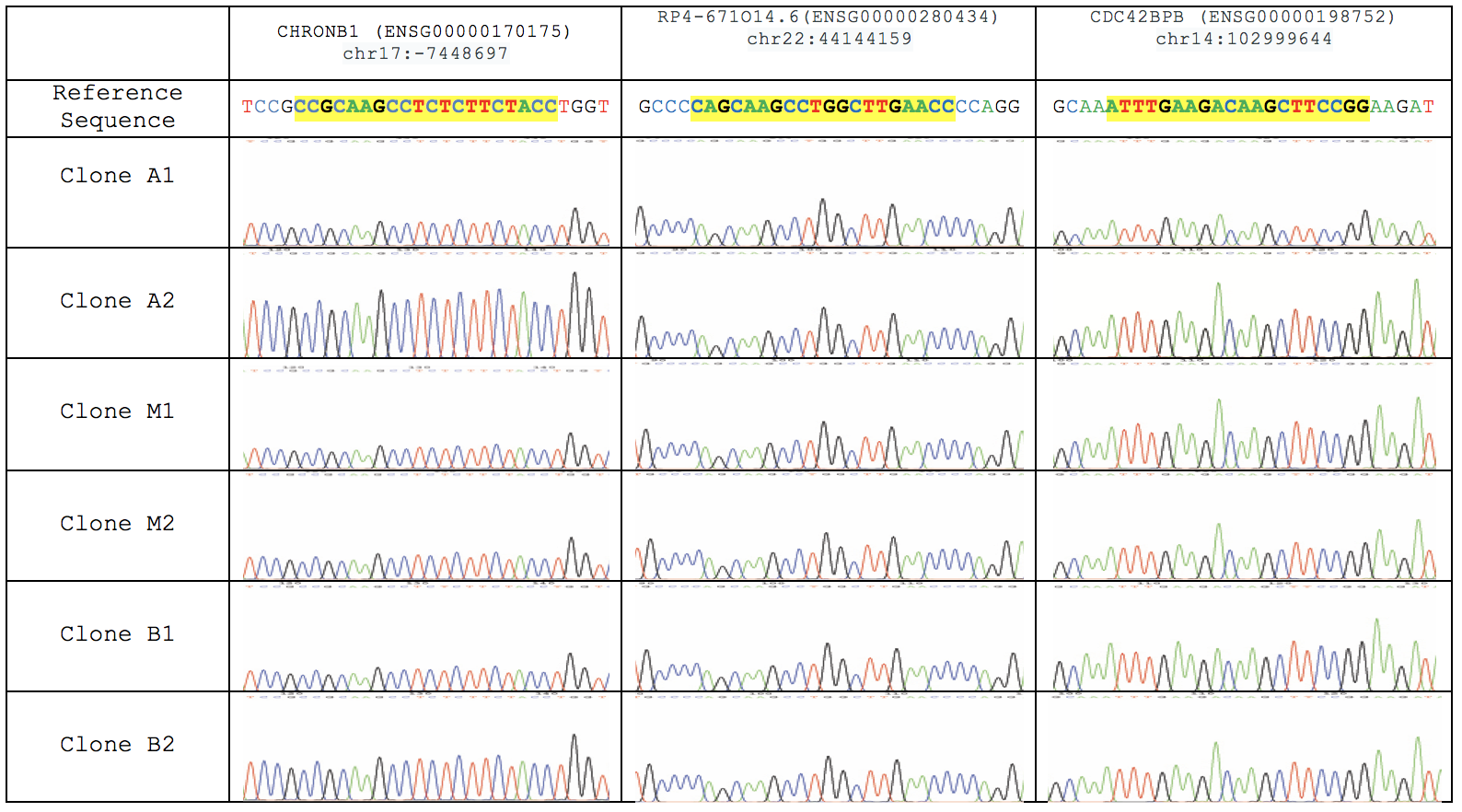

Supplement: S5 Fig — Using the CRISPR Design Tool at the Broad Institute, we were able to identify putative off target regions in the genes above. Primers flanking the putative off-target sites were used to amplify regions in CHRONB1, RP4-671014.6, and CDC42BPB, which were then analyzed via Sanger sequencing. All STAT6-/- clones did not exhibit any mutations, indicating that no off-targeting was present in these clones. (DOCX) [file pone.0234146.s005.docx]
