# Supplementary material for: Loss of STAT6 leads to anchorage-independent growth and trastuzumab resistance in HER2+ breast cancer cells
Source: PLoS One. 2020 Jun 11;15(6):e0234146. doi: 10.1371/journal.pone.0234146 (PMC7289443; doi:10.1371/journal.pone.0234146)
Supplement: S1 File — (DOCX) [file pone.0234146.s006.docx]

**Supplemental Results**

## Generation of *STAT6^-/-^* clones

Both alleles of STAT6 were subjected to CRISPR/Cas9-mediated somatic cell gene targeting. All clones (MCF-10A-derived clones A1 & A2, M15-derived clones M1 & M2, and BT474-derived clones B1 & B2) were isolated and whole cell protein lysates were used to confirm loss of the STAT6 protein (Fig 1 and S1).

A donor template was not used; therefore, random indels were introduced at the Cas9-mediated cleavage site via NHEJ. In order to determine the composition of each STAT6 allele, we used either next generation sequencing or TA cloning (S3 Fig). All clones underwent genetic alterations, which resulted in a frameshift. The clones either produced a premature stop codon shortly downstream and/or led to disruption of intron/exon borders.

The NGS results for clone M2 showed that only one variant was present. This suggests that either a homozygous deletion is present or a large deletion extending beyond one or both screening primers is present. Therefore, we PCR amplified a 1kb and 2kb region flanking the cut site and then visualized the product using gel electrophoresis and still failed to identify another variant (S4 Fig). Since our initial 500bp PCR product failed to show a second band with an indel and the larger amplicon failed to show a deletion, there is likely a homozygous deletion present. Sanger sequencing confirmed that gene targeting did not occur at any of the putative exonic off-target locations (S5 Fig).
