# Supplementary material for: Loss of STAT6 leads to anchorage-independent growth and trastuzumab resistance in HER2+ breast cancer cells
Source: PLoS One. 2020 Jun 11;15(6):e0234146. doi: 10.1371/journal.pone.0234146 (PMC7289443; doi:10.1371/journal.pone.0234146)
Supplement: S1 Raw Image — (PDF) [file pone.0234146.s007.pdf]

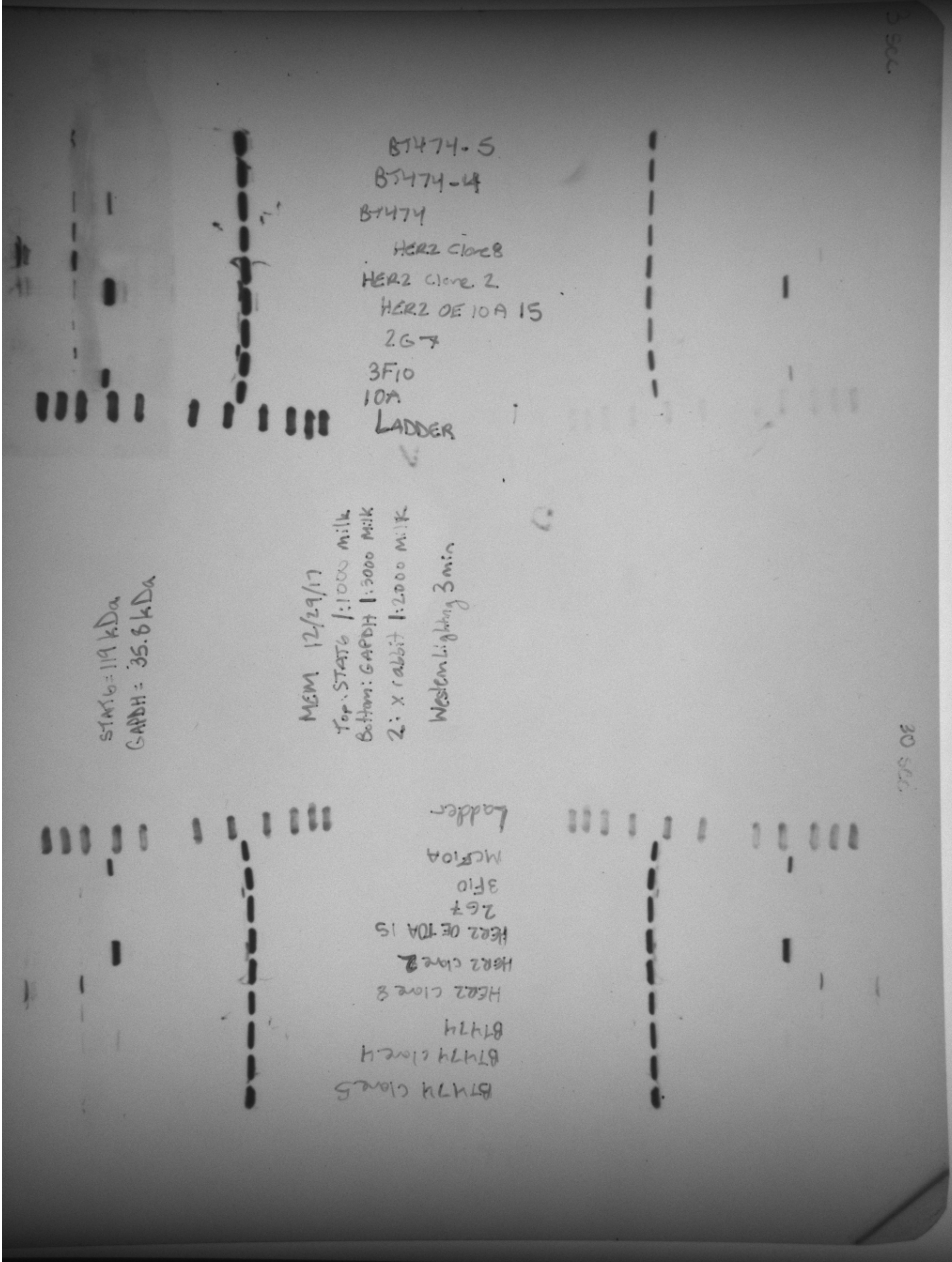
BT474-5
BT474-4
BT474
HER2 Clone8
HER2 Clone 2
HER2 OE 10A 15
2G7
3F10
10A
LADDER
STAT6 = 119 kDa
GAPDH = 35.6 kDa
MEM 12/29/17
Top: STAT6 1:1000 milk
Bottom: GAPDH 1:3000 milk
2°: x rabbit 1:2000 milk
WesternLighting 3 min
BT474 clone5
BT474 clone4
BT474
HER2 clone 8
HER2 clone 2
HER2 OE 10A 15
2G7
3F10
MCF10A
Ladder
30 sec
30 sec
